# Federated Ensemble Regression Using Classification

**DOI:** 10.1007/978-3-030-61527-7_22

**Published:** 2020-09-19

**Authors:** Oghenejokpeme I. Orhobor, Larisa N. Soldatova, Ross D. King

**Affiliations:** 8 University of Bari Aldo Moro, Bari, Italy; 9 Aristotle University of Thessaloniki, Thessaloniki, Greece; 10 Open University of Cyprus, Nicosia, Cyprus; 11 Dalhousie University, Halifax, NS Canada; 12 Department of Chemical Engineering and Biotechnology, University of Cambridge, Cambridge, CB3 0AS UK; 13 Department of Computing, Goldsmiths, University of London, London, SE14 6AD UK; 14 The Alan Turing Institute, London, NW1 2DB UK; 15 Department of Biology and Biological Engineering, Chalmers University of Technology, 412 96 Gothenburg, Sweden

**Keywords:** Ensemble learning, Machine learning, Regression, Bioinformatics, Gene expression

## Abstract

Ensemble learning has been shown to significantly improve predictive accuracy in a variety of machine learning problems. For a given predictive task, the goal of ensemble learning is to improve predictive accuracy by combining the predictive power of multiple models. In this paper, we present an ensemble learning algorithm for regression problems which leverages the distribution of the samples in a learning set to achieve improved performance. We apply the proposed algorithm to a problem in precision medicine where the goal is to predict drug perturbation effects on genes in cancer cell lines. The proposed approach significantly outperforms the base case.

## Introduction

In a standard regression setting, one builds a model on pre-existing learning data with the goal of making predictions on future unseen samples. In this case, a single model is built using a preferred learning algorithm. However, it has been demonstrated that one can improve predictive accuracy even further by aggregating the predictive power of multiple models built using the same learning data [14]. These models can be built in a variety of ways, from varying the attributes used in building the models to using multiple learning algorithms. This is done to ensure heterogeneity in the models, such that given a set of new samples, they are all wrong in different ways and their aggregation leads to improved predictions [5]. The typical approach in the use of a single model or an ensemble is that the distribution of the continuous response one is interested in predicting is often not given much thought. For example, if one imagines that the response for the samples in a dataset follows a normal distribution. Then it also follows that any model that is naively built using this data is going to be very good at predicting samples that a near the centre of the distribution, but not those at the tails. A close analogy to this phenomenon is the class imbalance problem in a classification setting. Where given a dataset in which one class is over-represented, models built on this dataset using machine learning algorithms typically perform poorly when presented with a sample from the under-represented class [2, 15]. Therefore, we hypothesised that predictive performance in a regression setting can be improved by accounting for the distribution of the response.

**Fig. 1.**
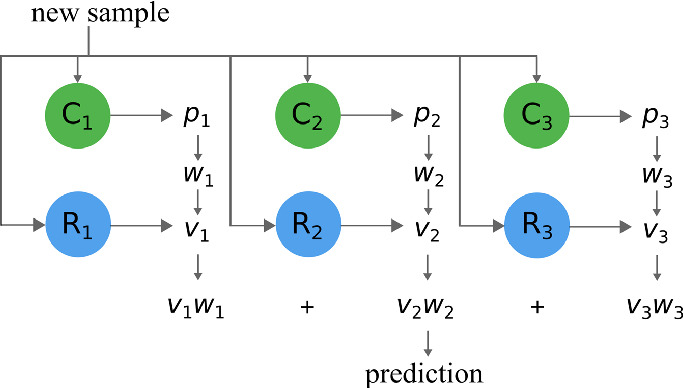
Representation of the proposed approach when bin size is 3.

We take an ensemble learning approach to solving this problem. First, we split the learning data into a pre-specified number of bins using a known discretization technique [9]. We then build a regressor for each bin using only the samples that belong to that bin, each of which generalises on only a restricted portion of the distribution. We then build a classifier for each bin, treating the samples which belong to said bin as the positive samples, and the samples in the other bins as the negative samples. Therefore, there is a classifier-regressor pair for each bin. Given an unseen sample, real-valued predictions are made using the regressor for each bin. The corresponding classifier for each regressor is then used to predict the probability that the unseen sample is similar to the samples used in building the regressor. The predictions are then aggregated by weighting the probabilities and applying them to the predictions. This process is described diagrammatically in Fig. 1.

This approach is valuable to problems in precision medicine, where tail case prediction is of vital importance. An example of such a problem is the prediction of drug perturbation effects on genes in cancer cell lines, which, with improved predictive accuracy, has the potential to dramatically improve the rate at which new cancer drugs are developed. In our evaluation, we used data from the library of integrated network-based cellular signatures (LINCS) [16], which curates the drug perturbation effects on human genes. Our evaluation shows a significant improvement in performance over the base case. Our contributions are as follows:

1. An ensemble learning approach which considers response distribution for regression problems.
2. An application to a real-world dataset in precision medicine.

## Related Work

Ensemble learning takes a variety of forms, from bootstrap aggregating (bagging) which is central to popular and robust learning algorithms like random forests [4], to methods like stacking [3]. The proposed approach shares some similarities with both of these methods. Stacking is most commonly used when one intends on aggregating the predictions made by multiple learning algorithms, or if a single learning algorithm is used, multiple models are built using subsets of the feature space [18]. There are three main processes in a stacking procedure: meta-feature generation, pruning, and aggregation [17]. Assume one has a learning and a test set. In the meta-feature generation phase, meta-features are generated for both the learning and test sets, and total to the number of models whose predictive power one wants to aggregate. Pruning is then used to optimise for the best meta-features. Finally, aggregation is done by learning weights using the learning set meta-features and then applying these weights to the test meta-features to form the final prediction.

In contrast to a typical stacking approach which we have described, we do not generate meta-features in our approach. The utility of the meta-features is that they provide a mechanism through which aggregating weights can be learned using a meta-level learning algorithm. Instead, we opt for a scheme where given a new sample, individual classifiers predict how much we can trust the predictions of their corresponding regressor as described in the introduction, which is more closely aligned with the concept of local classifiers in the hierarchical classification literature [20]. This implies that we also do not perform a pruning step. It is worth noting that while aggregation in stacking can be performed using weights learned with a meta-learner, it is also possible to simply average the predictions, we explore this in our evaluation. Other similarities exist. For example, one can argue that our weighting and aggregating procedure is a form of dynamic weighting, where new samples are weighted based on their similarities to samples used in building a model [19]. However, rather than being a separate step, dynamic weighting is implicit in the proposed learning procedure.

Central to the proposed method is the discretization of the continuous response one is interested in learning how to predict. Several methods to perform this task have been proposed, and they have been classed into supervised and unsupervised methods [7, 9]. We considered only unsupervised methods in our evaluation. However, the use of supervised methods will be explored in future work. Methods which use classification as a means to perform regression in an ensemble setting have also been proposed. Ahmad et al. proposed the use of extreme randomized discretization to perform regression via classification [12]. In contrast to what we propose, the authors do not use a classifier-regressor pair to estimate the prediction for a new sample. Rather they do this using the minimum or maximum of the training data points and the bin boundary [1, 12]. Also closely related to what we propose is work by Gonzalez et al. for problems that involve multi-variate spatio-temporal data [11]. The main differences in our approaches is two-fold. Firstly, they are interested in classifying bands of attributes before performing regression. Secondly, aggregation is done by first selecting the best models using leave-one-out cross-validation and the median predicted values by these models is treated as the final prediction for a new sample.

## Methodology

### Algorithm

The proposed approach can be split into a training and a prediction phase. An informal description follows, however, a more formal representation is given in Algorithm 1. In the training phase, given a training set with input vectors, a response, and a pre-specified number of bins *c*:

1. Discretize the response into *c* bins, forming *c* datasets.
2. For each *c* bin, build a regressor $R_c$ and a classifier $C_c$. The regressor is built using the training samples for the particular bin. Whereas, the classifier is built by treating the samples in the current bin as the positive class and all other samples in the training set as the negative class.

In the prediction phase, given a new sample:

1. With all the $R_c$ regressors, predict values for the new sample.
2. With all the $C_c$ classifiers, predict the probability that the sample belongs in that *c* bin.
3. Generate weights using the *c* probabilities such that they sum to 1. This is done by summing the *c* probabilities and then dividing each *c* probability by this sum.
4. Get the final prediction by summing the values generated by applying each corresponding *c* weight to the prediction made by its *c* regressor in step (1).

### Considerations

When tackling a machine learning problem, the choice of learning algorithm is vital as it plays a crucial role in predictive performance. However, it is clear from the description of the proposed approach outlined above that it is learner agnostic. That is, one can choose to build the classifiers and regressors using their preferred algorithm of choice. This property is particularly useful as one can choose to optimise for different properties using approaches from multiple kernel learning [22] or even stack multiple learning algorithms if they so choose. The choice of discretization technique is also open-ended, where one can choose to use known supervised or unsupervised discretization techniques, or a custom technique tailored to a particular problem.

When the number of bins is greater two, it will generally be the case that there will be some form of class imbalance. This may be in favour of the positive or negative class, and can be quite severe, depending on the distribution of the response variable under consideration, choice of discretization technique, and the number of specified bins. Therefore, it is important that this be taken into consideration, as it is known that class imbalance can have significant effects on predictive accuracy [2]. To combat this, methods which balance an imbalanced dataset such as oversampling methods like the synthetic minority oversampling technique (SMOTE) [6] should be considered. We explore the effects of discretization technique and class imbalance in our evaluation.
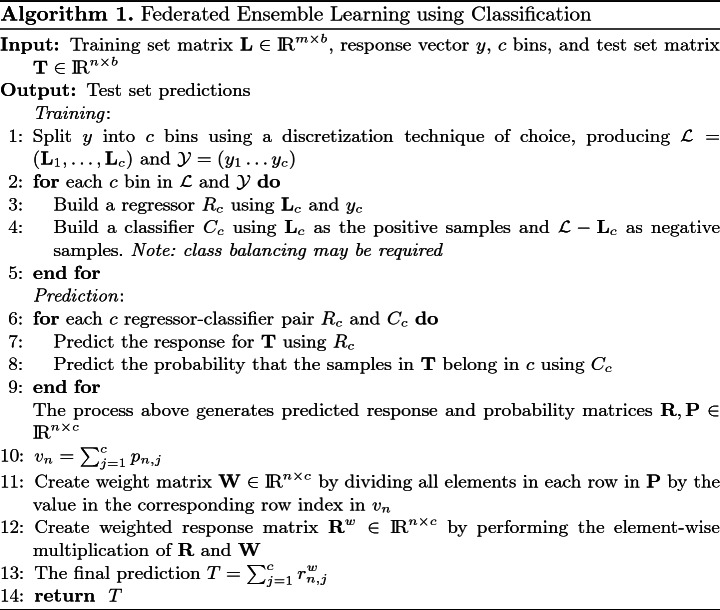


## Evaluation Setup

We used data from the general LINCS Phase II dataset with accession code GSE70138. We had 7000 training samples and 3000 test samples. The predictive task is the expression levels of 20 cancer-related genes [8, 10] using perturbation conditions as input. We evaluated four bin sizes: 2,3,4, and 5. We also considered four discretization methods. The first involves randomly assigning samples to bins, the second involves splitting samples evenly into bins after sorting, the third and fourth are equal frequency interval and k-means clustering. It worth noting that even splitting and frequency interval are the same in that they discretize a vector of continuous variables evenly given a specified size. However, they differ in that equal frequency does not achieve perfect equally sized groups if there are duplicates, naive even splitting does. For aggregation methods, we considered simple averaging, a case in which no classifiers are used in aggregation. For the cases in which classifiers are involved, we considered one in which class imbalance is ignored, we refer to this simply as imbalanced for the rest of the manuscript. The other classifier approaches used are one in which undersampling is used to reduce the number of samples in one class when it outweighs those in the other, and oversampling, which is the reverse. Undersampling was performed by randomly selecting samples from the over-represented class equal to that of the under-represented class. Oversampling was performed using SMOTE with the smotefamily package [21], where $k=5$. We used random forests as our learning algorithm. All models were built using 1000 trees and default settings with the ranger [24] library in R [13]. The reported performance metric for regression is the coefficient of determination ($R^2$), as we are interested in the amount of the observed variance explained by the ensemble. We also report the performance of the classifying aggregators, for these we report accuracy, precision, recall and the F1 score. The dataset used in our experiment is available here http://dx.doi.org/10.17632/8mgyb6dyxv.2, and it is named *base_fp*, and the code is available here https://www.github.com/oghenejokpeme/FERUC.

## Results

### Overall Performance

We observed that on average multiple combinations of the considered discretizer-aggregator pairs generally outperformed the base case (see Table 1). Certain discretizer-aggregator pairs tended to consistently perform well or poorly. Even split and frequency interval combined with oversampling outperformed all other combination pairs, whereas k-means combined with averaging or undersampling generally underperformed when compared to the others (see Table 2). When paired with oversampling, the even split and frequency interval discretizers both achieved an average percentage performance increase of approximately $100\%$ over the base case. Combined, both of these methods performed best when the number of bins is set to 5 (Table 3). With the assumption that there is no difference in performance between these two combinations and the base case, paired t-tests suggest that the null hypothesis can be rejected with a significance level of 0.01, with p-values of $2.6\times 10^{-8}$ and $9.7\times 10^{-9}$ respectively. Note that the average percentage performance difference between two competing approaches is calculated by estimating the percentage difference in performance for each gene pair, and then finding the mean.

**Table 1.** Mean predictive performance ($R^2$) of the 20 considered cancer genes for bin sizes 2, 3, 4, and 5. Discretization methods: random, even split, frequency interval, and k-means. Aggregation methods: simple averaging (AVG), class imbalance is ignored (RG), undersampling (US), and oversampling (OS). The best performing method for each bin size is underlined.

*Bins*	*Base*	Random				Even Split				Frequency Interval				K-means			
		AVG	RG	US	OS	AVG	RG	US	OS	AVG	RG	US	OS	AVG	RG	US	OS
2	0.075	0.079	0.079	0.079	0.079	0.057	0.081	0.081	0.081	0.057	0.082	0.082	0.082	−0.496	0.080	−0.451	0.054
3	0.075	0.081	0.083	0.083	0.085	0.040	0.085	0.088	0.100	0.040	0.085	0.086	0.094	−0.678	0.082	−0.500	0.050
4	0.075	0.082	0.084	0.084	0.088	0.031	0.086	0.084	0.103	0.031	0.086	0.084	0.102	−0.567	0.081	−0.432	0.036
5	0.075	0.082	0.084	0.082	0.091	0.024	0.087	0.078	0.103	0.024	0.087	0.078	0.104	−0.433	0.082	−0.302	0.031

**Table 2.** Average percentage performance difference of the two best and worst performing discretizer-aggregator combinations compared to the base case for each bin. The percentage increase or decrease is given, followed by the number of genes for which a discretizer-aggregator pair outperforms the base case. The best and worst performers are in boldface.

*Bin*	Best performers	Worst performers
2	Frequency interval – oversampling	k-means – undersampling
	$26.5\% (17)$	$-1324.2\% (3)$
	Frequency interval – imbalanced	k-means – averaging
	$26.4\% (17)$	$-1289.0\% (1)$
3	Even split – oversampling	**k-means – averaging**
	$105.5\% (20)$	${\varvec{-1732.5\% (0)}}$
	Frequency interval – oversampling	k-means – undersampling
	$75.3\% (20)$	$-1460.3\% (0)$
4	**Even split – oversampling**	k-means – averaging
	${\varvec{118.3\% (20)}}$	$-1643.3\% (1)$
	Frequency interval – oversampling	k-means – undersampling
	$113.4\% (20)$	$-1520.4\% (0)$
5	Even split – oversampling	k-means – averaging
	$117.5\% (20)$	$-856.5\% (1)$
	Frequency interval – oversampling	k-means – undersampling
	$116.7\% (20)$	$-864.2\% (0)$

**Table 3.** Predictive performance ($R^2$) of the considered genes when bin size is 5. Shown are the results for the base case, the even split – oversampling pair (ES–OS), and frequency interval – oversampling pair (FRQ–OS). The percentage increase over the base case is also given.

*Genes*	*Base*	ES–OS	FRQ–OS
AKT1	0.197	0.207 (5.1)	0.208 (5.6)
APOE	0.071	0.088 (23.9)	0.090 (26.8)
BRCA1	0.111	0.152 (36.9)	0.152 (36.9)
CDH3	0.032	0.056 (75.0)	0.058 (81.2)
CDK4	0.291	0.294 (1.0)	0.296 (1.7)
CFLAR	0.017	0.049 (188.2)	0.049 (188.2)
EGF	0.055	0.076 (38.2)	0.076 (38.2)
EGFR	0.051	0.090 (76.5)	0.089 (74.5)
FGFR2	0.059	0.086 (45.8)	0.085 (44.1)
IGF1R	−0.007	0.042 (700.0)	0.041 (685.7)
KIT	0.069	0.096 (39.1)	0.098 (42.0)
LYN	0.083	0.109 (31.3)	0.112 (34.9)
PAX8	0.011	0.041 (272.7)	0.040 (263.6)
PTK2	0.069	0.082 (18.8)	0.082 (18.8)
RAD51C	0.057	0.087 (52.6)	0.087 (52.6)
STK10	0.044	0.066 (50.0)	0.067 (52.3)
TERT	0.073	0.107 (46.6)	0.108 (47.9)
TGFBR2	0.010	0.045 (350.0)	0.044 (340.0)
TNFRSF21	−0.022	0.042 (290.9)	0.042 (290.9)
TP53	0.228	0.245 (7.5)	0.247 (8.3)

### Discretizer Effects

**Fig. 2.**
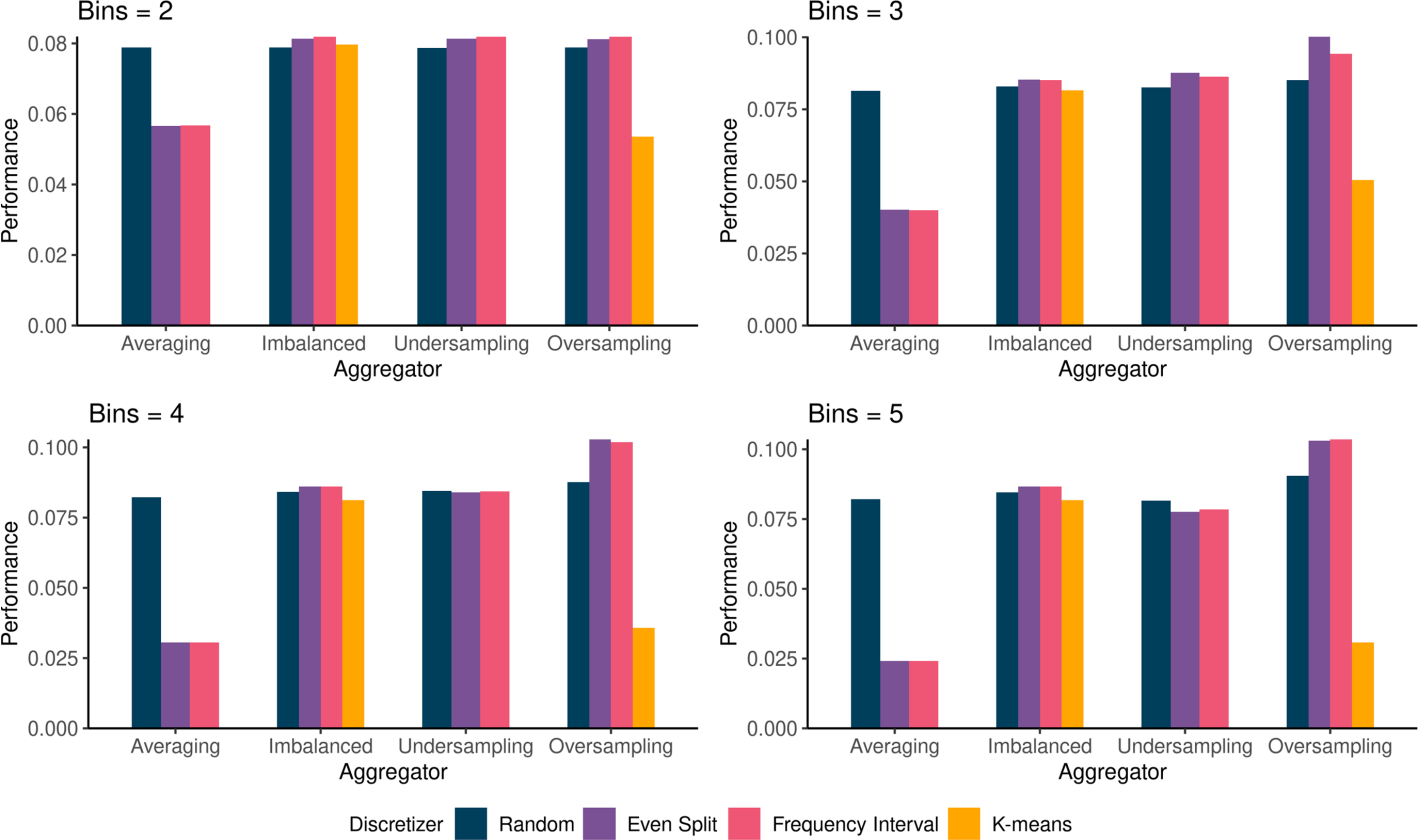
Average discretizer performance ($R^2$) for the considered bin sizes across the considered aggregation approaches. Frequency interval is excluded from averaging and undersampling aggregation results because it consistently produced negative $R^2$ values.

Discretization is the first step in the proposed learning algorithm, and the method by which we stratify the distribution of the response one might be interested in predicting into narrow-bins (Algorithm 1). It is clear from Fig. 2 that the choice of discretizer plays a crucial role in predictive performance. When averaging is used as the aggregator, random sampling outperforms all other discretizers, with even split and frequency interval performing equally well. This is interesting as it shows that without the aggregating classifiers, the regressors built using methods like frequency interval perform worse than those built using random sampling. The reason is because when the response is put into bins using random sampling, the values in each of these bins will generally follow the same distribution as the overall response. Therefore, aggregating the predictions made by regressors built using these bins by averaging will generally yield good results. This is in contrast to when methods like even split or frequency interval are used, as each bin comprises of narrow generally non-intersecting bands of the overall distribution. It is worth noting here though that k-means performs remarkably poorly, producing negative $R^2$ values, suggesting that it fits worse than the horizontal line. One might be quick to note that this is one of the disadvantages of using $R^2$ as a performance metric in a regression problem when there is the potential for non-linearity. However, we would argue that for this particular application, it is vital that we have a clear representation of how much of the observed variance is explained by the proposed ensemble.

When class imbalance is ignored as is the case in the imbalanced aggregators, we observed that even split and frequency interval have near identical performance, with k-means and random sampling coming third and fourth depending on bin size. When undersampling is used to balance the dataset before building the classifying aggregators, we observed that as the number of bins increases, random sampling tended to outperform the even split and frequency interval discretizers. This is because as bin size increases, the number of samples in each bin decreases, and by undersampling, the classifying aggregators are built using fewer and fewer samples, making them less powerful. The performance of the random sampling discretizer does not suffer as much from this because its regressors are built using bins which generally represent the overall distribution of the response. We discuss this further when we discuss aggregator effects in the next section.

The performance of the discretizers when oversampling is used to handle class imbalance supports and contrasts with their performance when undersampling is used. We observed that the even split and frequency interval discretizers generally perform vastly better than how they do when undersampling is used to deal with class imbalance. In contrast to undersampling, the classifying aggregators are built using datasets in which the positive class has been oversampled, improving the models which classify new samples into bins. Given that the overall distribution of a response is represented in each bin when random sampling is used, building accurate bin delineating classifiers becomes more difficult as the samples in the positive and negative classes are very much alike. However, the expectation is that these classifiers will essentially predict that a new sample belongs in its bin, and produce a probability based on how closely related it is to the positive samples used in their construction. Therefore, for the random sampling discretizer, one would expect better performance when undersampling is used, which is what we observed (see Fig. 2).

### Aggregator Effects

In the previous section, we discussed the effects the choice of discretizer can have on predictive performance. Although the discretizers were our main focus, it is clear that there is a synergistic effect between the choice of discretizer and aggregator. Figure 2 also shows that the choice of aggregator has a clear effect on predictive performance, with averaging performing worse overall, oversampling outperforming all the others, and undersampling generally performing worse than imbalanced. Here, our primary focus is to discuss why this is the case, especially as it has to do with the classifying aggregators. Table 4 shows the average predictive performance (accuracy, precision, recall, and F1 score) for all discretizer-aggregator pairs, and for all bin sizes we considered. These results explain the observed predictive performance discussed in the previous two sections. Although the accuracy of the classifying models are also reported, our discussion will be mostly centered around the precision and recall metrics, given that we are dealing with input datasets which may be class imbalanced.

**Table 4.** Average predictive performance of the aggregating classifiers built using datasets whose class representations are imbalanced (RG), undersampled (US), and oversampled (OS) for the considered discretizers. The reported performance metrics are accuracy (Acc), precision (Prec), recall (Rec), and F1 score.

*Discretizer*	*Aggregators*	Bins															
		2				3				4				5			
		Acc	Prec	Rec	F1	Acc	Prec	Rec	F1	Acc	Prec	Rec	F1	Acc	Prec	Rec	F1
Random	RG	0.50	0.50	1.00	0.67	0.13	0.13	1.00	0.23	0.05	0.05	1.00	0.09	0.02	0.02	1.00	0.03
	US	0.50	0.50	1.00	0.66	0.50	0.50	1.00	0.67	0.50	0.50	1.00	0.67	0.50	0.50	1.00	0.67
	OS	0.50	0.50	1.00	0.67	0.17	0.17	1.00	0.29	0.10	0.10	1.00	0.18	0.06	0.06	1.00	0.11
Even split	RG	0.58	0.58	0.58	0.58	0.65	0.23	0.45	0.30	0.73	0.10	0.37	0.16	0.79	0.06	0.32	0.10
	US	0.58	0.58	0.58	0.58	0.56	0.56	0.39	0.46	0.55	0.55	0.29	0.38	0.54	0.54	0.23	0.32
	OS	0.58	0.57	0.58	0.58	0.65	0.26	0.44	0.33	0.72	0.17	0.35	0.22	0.77	0.12	0.30	0.17
Frequency interval	RG	0.58	0.58	0.58	0.58	0.65	0.23	0.45	0.30	0.73	0.10	0.37	0.16	0.79	0.06	0.32	0.10
	US	0.58	0.58	0.58	0.58	0.56	0.56	0.39	0.46	0.55	0.55	0.29	0.38	0.54	0.54	0.23	0.32
	OS	0.58	0.58	0.58	0.58	0.65	0.25	0.44	0.32	0.72	0.15	0.36	0.21	0.77	0.12	0.30	0.16
K-means	RG	0.80	0.55	0.67	0.55	0.73	0.36	0.52	0.34	0.77	0.24	0.37	0.15	0.80	0.15	0.15	0.06
	US	0.60	0.60	0.55	0.52	0.60	0.60	0.39	0.42	0.60	0.60	0.30	0.34	0.60	0.60	0.24	0.29
	OS	0.79	0.58	0.62	0.58	0.73	0.42	0.47	0.43	0.76	0.32	0.39	0.34	0.79	0.25	0.33	0.27

When random sampling the discretizer, we observed that across all bin sizes, the recall of all the classifying aggregators is exactly 1. This is consistent with previously discussed results. It shows that the classifiers are classifying all the test samples as being similar to those used in their building. This is unsurprising since the samples used in building each bin’s classifier follows the same distribution as the original response vector. The precision of the aggregating methods is more nuanced. In the case in which class imbalance is ignored, although the recall maintains its value of 1 as bin size increases, the precision steadily decreases. This makes sense, as the expectation is that the models will consistently become worse at identifying false positives. The results for undersampling and oversampling are contrasting. While recall is also consistently 1 as bin size increases, the precision for undersampling stays at approximately 50%, while like the class imbalance case, the precision for oversampling steadily declines. This is also consistent with expectation. In the case of undersampling, we are building binary bin classifiers using a perfect 50−50 split in class representation, but with fewer samples as bin size increases. It is no surprise that accuracy is also approximately 50%. For oversampling, accuracy and precision both hold 50% when bin size is 2, but steadily declines as it increases. Here, we argue that oversampling the samples in the imbalanced class, which is usually the positive class, makes the classifiers even worse at predicting false positives. This is to be expected, due to the properties of the random sampling discretizer.

For the even split and equal frequency discretizers, all three aggregators have an average value of 58% for accuracy, precision, recall, and F1 when the bin size is 2. This suggests that the models are capable of classifying positive and negative samples equally well. However, this changes as bin size increases. When the input dataset is imbalanced, we observed that both precision and recall steadily decreases, with precision getting remarkably worse-off than recall. The explanation for this is that the class imbalance is exacerbated by the increasing bin size with fewer samples in each bin, making it harder for the models to identify false positives. When undersampling is used, precision generally remains the same as bin size increases but recall decreases. This shows that while the classifiers’ false positive prediction rate does not get significantly worse, its number of false negative predictions increases. This phenomenon can be easily explained by the fact that as bin size increases, fewer samples in general are used in building the classifiers. For oversampling, what we observe for recall and precision are in contrast to those of undersampling. Though they both decrease as bin size increases, recall is better than precision. When compared to the imbalanced case, although the recall values are similar, the precision in the oversampling case is generally better, especially as bin size increases. This explains why oversampling outperforms the imbalanced and undersampling cases. The difference in performance between even split and frequency interval as seen in Table 2 can be explained by a slight increase in precision and a slight decrease in recall for even split compared to frequency interval (see Table 4). Therefore, it is worth noting that for the proposed approach, seemingly duplicate values should not be excluded during discretization.

For k-means, the precision and recall values are generally similar to those of even split and frequency interval for the considered bin sizes with the exception of when bin size is 2. However, even with this similarity, we still observed that the undersampling aggregator performed remarkably poorly when paired with k-means (see Tables 1 and 2). Our analysis of the results showed that this is because of a known limitation of k-means discretization, which is that it is very sensitive to outliers [7], which we expect to mainly be at the tails of the response distribution. Individual investigation of classifier performance for each gene showed that this occurs because the models built using samples at the tails have either very low precision and very high recall, or the opposite. This is in contrast to other discretizers, for which the precision-recall ratio is better balanced. This is evident from the difference in F1 scores between the different discretizers across the considered bin sizes (see Table 4).

**Fig. 3.**
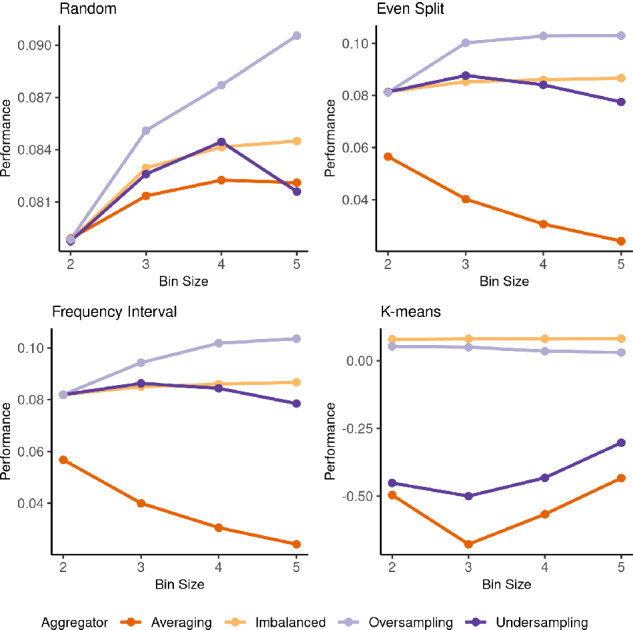
Average aggregator performance ($R^2$) for the considered discretizers as bin size increases.

### Bin Size Effects

Figure 3 shows how aggregator predictive performance changes as bin size increases for the considered discretizers. For the random discretizer, most aggregators tend to steadily improve as bin size increases. The exception to this is undersampling, which peaks at a bin size of 4. For even split and frequency interval, the four aggregators behave similarly as expected. The imbalanced and oversampling aggregators get better as bin sizes increases, with the imbalanced aggregator doing so at a slower rate. Averaging gets worse as bin size increases as discussed in previous sections. Lastly, the undersampled aggregators reach peak performance at a bin size of 3 and begin to decline. When the k-means discretizer is paired with averaging and undersampling, we see a performance decrease from bin size 2 to 3, then steady increase from 3 to 5. However, as noted in the previous two sections, the performance is still remarkably poor. For oversampling, predictive performance sees a slow decline as bin size increases. Whereas the imbalanced aggregator tends to hold its performance. From these results, it is clear that bin size also plays a crucial role in the performance of the proposed ensemble regression approach. However, to what extent this is the case is beyond the scope of this work and will be the subject of future work.

## Discussion

An important task in the machine learning model building process is the selection of the right parameters. Our results show that the choice of bin size, discretizer, and aggregator all play an important role in predictive performance. Although we do not directly evaluate it here, we argue that these parameters can be easily optimised using the standard model selection approach with cross-validation. Assuming a near optimal bin size has been selected, the proposed ensemble learning algorithm is limited by the fact that it can only do as well the classifier-regressor pairs. Although we used only random forests in our evaluation, which is capable of building both classifiers and regressors, one can choose to use one learning algorithm for the classifiers and another for the regressors. In fact, it is possible to extend what we have proposed using traditional stacking, where multiple learning algorithms are used as classifiers and regressors. Of course this will come with increased cost in the form of computational time complexity. Another obvious extension is in multi-target regression problems. For example, one can imagine using this as the core predictor in an ensemble of regressor chains [23]. All of this, along with evaluations on other datasets will be the subject of future work.

## Conclusion

We have presented an ensemble learning algorithm for regression using classification which leverages the underlying distribution of the response one is interested in predicting. We evaluated this approach on an important problem in precision medicine, which is the *in silico* estimation of drug perturbation effects on genes in cancer cell lines. We found that this approach significantly outperforms the base case, with several directions for extension which we conjecture will further improve its predictive capabilities.
